# Ultrasound-Guided Needle Aspiration Biopsy for Superficial Cervical Lymphadenopathy: Diagnostic Yield Beyond Tuberculosis

**DOI:** 10.3390/diagnostics16183052

**Published:** 2026-09-20

**Authors:** Maria Angela Licata, Giorgio Monteleone, Enrico Schiavi, Virginia Di Bari, Annelisa Mastrobattista, Alberto Zolezzi, Serena Maria Carli, Carla Nisii, Daniele Colombo, Fabrizio Palmieri, Gina Gualano, Paola Mencarini

**Affiliations:** 1 Pulmonology Unit and UTIR, Ospedale Civile San Salvatore, 67100 L’Aquila, Italy; mav.licata@gmail.com; 2 Division of Respiratory Medicine, Department PROMISE, “Paolo Giaccone” University Hospital, 90100 Palermo, Italy; giorgio.monteleone@stud.uni-due.de; 3 Center for Interstitial and Rare Lung Diseases, Pneumology Department, Ruhrlandklinik, University Hospital Essen, University of Duisburg-Essen, 45147 Essen, Germany; 4 Department of Public Health and Infectious Diseases, Sapienza University of Rome, AOU Policlinico Umberto I, 00185 Rome, Italy; enrico.schiavi@uniroma1.it; 5 Respiratory Infectious Diseases Unit, National Institute for Infectious Diseases “Lazzaro Spallanzani” IRCCS, 00149 Rome, Italy; a.mastrobattista@inmi.it (A.M.); alberto.zolezzi@inmi.it (A.Z.); fabrizio.palmieri@inmi.it (F.P.); gina.gualano@inmi.it (G.G.); paola.mencarini@inmi.it (P.M.); 6 Infectious Diseases Unit, Ospedale Infermi, 47923 Rimini, Italy; serenamaria.carli@auslromagna.it; 7 Laboratory of Microbiology and Biorepository, National Institute for Infectious Diseases “Lazzaro Spallanzani” IRCCS, 00149 Rome, Italy; carla.nisii@inmi.it; 8 Pathology Unit, National Institute for Infectious Diseases “Lazzaro Spallanzani” IRCCS, 00149 Rome, Italy

**Keywords:** cervical lymphadenopathy, ultrasound-guided needle aspiration biopsy (US-NAB), modified Menghini aspiration needle, extrapulmonary tuberculosis, tuberculous lymphadenitis

## Abstract

**Background:** Superficial cervical lymphadenopathy frequently requires tissue sampling because infectious, inflammatory, and malignant diseases often share overlapping clinical and radiological features. Although ultrasound-guided sampling of superficial cervical lymph nodes is well established, the available literature predominantly focuses on fine-needle aspiration cytology (FNAC) or core needle biopsy (CNB). Comparatively less data specifically describe aspiration-based tissue acquisition providing material for combined histopathological, microbiological, and molecular analyses, particularly in very small cervical lymph nodes. We describe our single-center experience with ultrasound needle aspiration biopsy (US-NAB), focusing on technical feasibility, sample adequacy, diagnostic contribution, and procedural safety. **Methods:** We retrospectively analyzed 20 consecutive patients referred to a tertiary respiratory infectious diseases center between April 2023 and June 2024 for suspected cervical tuberculous lymphadenopathy. All patients underwent real-time US-NAB using an 18-gauge modified Menghini aspiration needle. Biopsy specimens were processed, as applicable, for histopathology, acid-fast bacilli smear microscopy, molecular testing (real-time PCR), and mycobacterial culture. Diagnostic yield, final diagnoses, lymph node size, and procedural safety were evaluated. **Results:** A final diagnosis of tuberculosis was established in 13 patients (65%), while seven patients (35%) received alternative diagnoses, including metastatic malignancies (20%), sarcoidosis (10%), and bacterial lymphadenitis (5%). Adequate material contributing to a definitive diagnosis was obtained in all 20 procedures (20/20). Notably, 60% of sampled lymph nodes measured <15 mm in short-axis diameter, including two nodes measuring only 5–6 mm. Histopathology was diagnostic of tuberculosis in eight patients, whereas five additional cases with nonspecific necrosis were confirmed by positive TB-PCR. No major procedure-related complications, including bleeding, pneumothorax, or pneumomediastinum, were observed. **Conclusions:** In this small single-center cohort, US-NAB was feasible and safe and provided adequate material for histopathological, microbiological, and molecular analyses, including when very small superficial lymph nodes were sampled. Beyond confirming tuberculosis, the procedure contributed to the identification of alternative benign and malignant diagnoses. These findings support further evaluation of US-NAB as a complementary sampling option in the diagnostic work-up of superficial cervical lymphadenopathy.

## 1. Introduction

Cervical lymphadenopathy represents a frequent clinical challenge because benign inflammatory diseases, tuberculosis, lymphoma and metastatic malignancies often share overlapping clinical and imaging features. Histological confirmation is therefore frequently required to establish a definitive diagnosis and guide patient management [1].

Cervical lymphadenopathy is the most common presentation of extrapulmonary tuberculosis (EPTB), accounting for 30–50% of EPTB cases worldwide, particularly in countries with a high disease burden. Tuberculous lymphadenitis (TL) often presents as unilateral, painless lymph node enlargement, but its clinical and radiological features can mimic other infectious, inflammatory, or neoplastic conditions, complicating timely diagnosis and prompt treatment initiation [1].

Historically, fine-needle aspiration cytology (FNAC) has been widely used for the diagnosis of TL due to its minimally invasive nature and relatively high specificity [2]. However, FNAC may provide limited material for combined cytological, microbiological, and molecular analyses in some cases, particularly when the aspirate is paucicellular or when multiple ancillary tests are required [2].

To overcome these limitations, ultrasound-guided core needle biopsy (US-CNB) has increasingly been adopted. By retrieving an intact tissue core, US-CNB provides preserved tissue architecture and generally improves the diagnostic yield for histopathological, microbiological, and molecular analyses [3,4]. Likewise, contrast-enhanced ultrasound-guided core needle biopsy (CEUS-CNB) may further improve lesion targeting, particularly in partially necrotic lymph nodes, thereby increasing diagnostic accuracy [5,6]. Compared with surgical excision biopsy, US-CNB is less invasive, more cost-effective, and can be safely performed in an outpatient setting [3,7].

Taken together, current evidence supports the use of US-guided core biopsy—ideally coupled with histopathology and rapid molecular diagnostics—as an effective diagnostic approach in selected patients with suspected cervical lymphadenopathy, providing material suitable for histopathological and molecular analyses [8,9]. This multimodal strategy enables rapid, accurate diagnosis and timely initiation of anti-tubercular therapy, ultimately improving patient outcomes [10].

Although the growing body of evidence supports the use of US-CNB in cervical tuberculous lymphadenitis, most studies investigating aspiration-based sampling have been conducted in the endoscopic setting [11]. Endosonography-guided tissue acquisition, including endobronchial ultrasound-guided transbronchial needle aspiration (EBUS-TBNA) and endoscopic ultrasound-guided fine-needle aspiration or biopsy (EUS-FNA/FNB), has demonstrated high diagnostic performance in mediastinal and abdominal tuberculous lymphadenopathy, particularly when cytological, microbiological, and molecular analyses are combined [11,12]. Ultrasound-guided sampling of superficial cervical lymph nodes is an established procedure in clinical practice. However, much of the available literature has focused on FNAC or core needle biopsy. Comparatively fewer reports have specifically described aspiration-based tissue acquisition using larger-bore aspiration needles to obtain material suitable for combined histopathological, microbiological, and molecular analyses.

An aspect of particular practical interest is the feasibility of aspiration-based sampling in very small superficial lymph nodes. Tissue acquisition from targets measuring less than 10 mm in short-axis diameter may be technically demanding and requires precise real-time visualization of the needle trajectory and tip.

Therefore, the aim of this study was to describe our single-center experience with US-NAB of superficial cervical lymph nodes, focusing on technical feasibility, sample adequacy, diagnostic contribution, and procedural safety, including its application to very small lymph nodes.

## 2. Materials and Methods

### 2.1. Study Design and Setting

This was a single-center, retrospective cohort study conducted at the National Institute for Infectious Diseases “Lazzaro Spallanzani” in Rome, Italy, a tertiary referral center for respiratory infectious diseases. Data were retrospectively collected from the medical records of patients evaluated between April 2023 and June 2024.

### 2.2. Patient Selection

We retrospectively collected data from 20 patients presenting with suspected tuberculous cervical lymphadenopathy—based on epidemiological risk factors, such as country of origin, positive QuantiFERON-TB Gold assay, and compatible clinical and radiological findings—who were admitted to the Institute during the study period. Although TB was the primary clinical suspicion, patients with alternative final diagnoses after biopsy were also included to describe the diagnostic contribution of the procedure across different conditions. The study included all consecutive patients with suspected cervical tuberculous lymphadenopathy who underwent US-NAB during the study period. No patient undergoing the procedure was selectively excluded from the analysis.

Available post-procedural clinical records were retrospectively reviewed to assess subsequent diagnostic confirmation or reclassification. Patients diagnosed with tuberculous lymphadenitis, sarcoidosis, or bacterial lymphadenitis remained under clinical follow-up at our center for approximately 12 months or more as part of routine clinical care. Patients with malignant diagnoses were subsequently referred to a dedicated oncology center for further management.

### 2.3. Ultrasound-Guided Biopsy Procedure

All ultrasound-guided needle aspiration biopsies (US-NAB) were performed by experienced respiratory physicians with expertise in thoracic ultrasound and interventional procedures. Patients were positioned supine with slight contralateral neck extension to optimize exposure of the target laterocervical region.

Real-time ultrasound examination was performed using an Esaote ultrasound system equipped with a high-frequency linear-array transducer (7–12 MHz). Before needle insertion, grayscale ultrasound was used to evaluate lymph node size, morphology, echogenicity, and the presence of necrotic areas, while color Doppler imaging was systematically employed to identify and avoid adjacent vascular structures and to define the safest needle trajectory.

After skin disinfection and sterile preparation of the procedural field, local anesthesia was achieved with 2% lidocaine infiltrated into the skin and subcutaneous tissues. Ultrasound-guided tissue sampling was then performed using an 18-gauge modified Menghini aspiration needle with a true free-hand technique, without the use of probe-mounted needle guides or other needle-guiding devices, under continuous real-time ultrasound guidance. The needle was advanced under direct visualization until its tip reached the target lymph node, allowing continuous monitoring throughout the procedure.

Once correctly positioned, the needle was moved back and forth within different areas of the lesion, with tissue aspiration generated by the intrinsic mechanism of the modified Menghini needle. Two needle passes were routinely performed for each target, with additional passes at the operator’s discretion when necessary to obtain sufficient material for histopathological, microbiological, and molecular analyses.

No minimum lymph node size was required for sampling. Target lymph nodes were selected according to their clinical relevance and sonographic characteristics, including cortical thickening, loss of the fatty hilum, heterogeneous echotexture, intranodal necrosis, or abnormal vascularization. When multiple pathological lymph nodes were identified, the most sonographically suspicious and technically accessible node was selected for sampling.

US-NAB was also performed in small superficial laterocervical lymph nodes with a short-axis diameter below 10 mm, including nodes measuring as little as 5 mm, when these findings raised suspicion of tuberculous involvement. In patients presenting with associated superficial collections suggestive of scrofula, the fluid component was sampled during the same procedure whenever technically feasible.

Immediately after collection, specimens were allocated, as applicable, for histopathological examination, acid-fast bacilli smear microscopy, mycobacterial culture, and molecular testing by real-time PCR, according to the institutional diagnostic protocol.

### 2.4. Sample Handling and Diagnostic Evaluation

The biopsy specimens obtained were subjected to histopathological examination, bacterioscopy, molecular testing, and identification of both tuberculous and nontuberculous mycobacteria by real-time PCR (Anyplex MTB/NTM real-time detection kit, Seegene, Seoul, Republic of Korea) and culture on standard Lowenstein–Jensen (LJ) medium.

For histopathological examination, the biopsy specimens were immediately fixed in neutral-buffered formalin and stored for 24 h before embedding in paraffin blocks. Tissue sections (3 μm thick) were stained with hematoxylin and eosin (H&E) for histopathological analysis. Deparaffinized and rehydrated sections were processed for Ziehl–Neelsen staining (Bio-Optica, Milano S.p.A, Milano, Italy), with an internal positive control, to detect acid-fast bacilli (AFB).

TB-PCR was performed in all patients. In five cases, the specimens obtained by US-NAB consisted predominantly of solid tissue; in these cases, priority was given to histopathological examination and molecular testing for *M. tuberculosis*. Direct smear microscopy was not prioritized in these predominantly solid specimens, as histopathological examination and molecular testing were considered more informative for the available material. Mycobacterial culture, although potentially providing additional microbiological information, was not performed in these five cases for organizational reasons.

A final diagnosis of tuberculous lymphadenitis was established on the basis of microbiological and/or compatible histopathological findings supported by immunological evidence. Cases with a positive molecular test and/or mycobacterial culture for *M. tuberculosis* were classified as microbiologically confirmed TB. In the absence of microbiological confirmation, a diagnosis of TB was based on histopathological findings consistent with tuberculosis together with a positive QuantiFERON-TB test.

### 2.5. Data Collection and Statistical Analysis

Demographic, clinical, radiological, and microbiological data were extracted from electronic medical records. Procedural safety, sample adequacy, diagnostic yield, and major procedure-related complications were retrospectively assessed from the available medical records.

For the purposes of this study, a sample was considered adequate when the material obtained was sufficient to perform the clinically indicated histopathological and/or microbiological and molecular investigations. No predefined quantitative tissue threshold was used.

Descriptive statistics were used to summarize the subject characteristics and the histopathological results. Continuous and categorical variables were reported as median [interquartile range (IQR)] and frequencies or percentages, respectively.

Due to the exploratory nature of the study and limited sample size, no formal hypothesis testing was conducted.

## 3. Results

Table 1 presents the demographic and clinical data of the 20 subjects, collected at the time of the procedure. Briefly, the patients had a median age of 46.5 years, and 55% were female. Among the 18 patients with available QuantiFERON-TB results, 14 (77.8%) tested positive. Significant comorbidities were present in 55% of patients, 95% were not considered immunocompromised, and 70% had a history of birth in or recent travel to tuberculosis-endemic countries.

**Figure 1:**
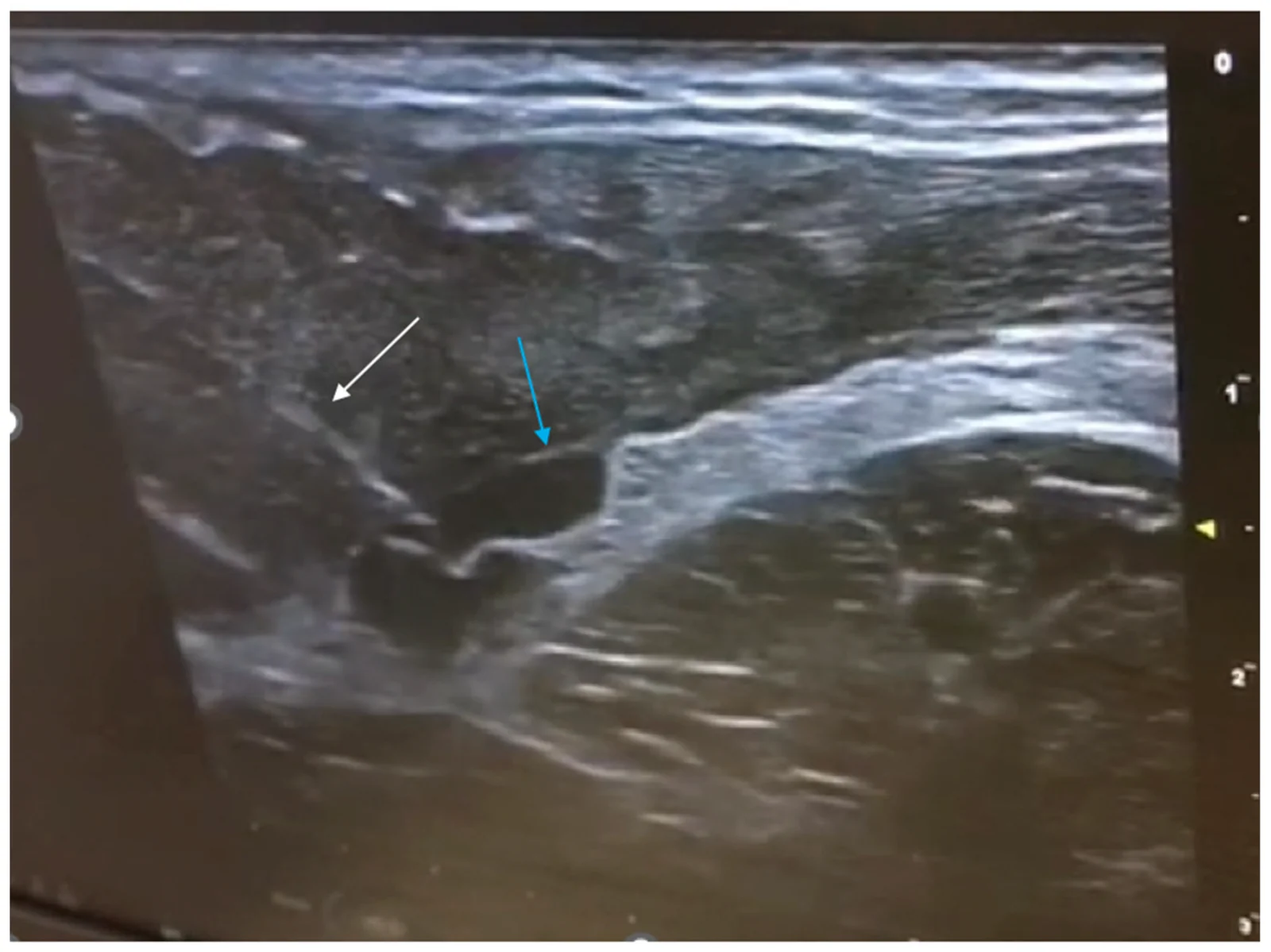
Ultrasound image of scrofula, showing a heterogeneous fluid-containing necrotic collection overlying a small laterocervical lymph node (blue arrow) measuring approximately 6 mm in short-axis diameter. The lymph node exhibits a preserved echogenic hilum. The biopsy needle (white arrow) is visualized with its tip positioned within the target lymph node under real-time ultrasound guidance.

**Figure 2:**
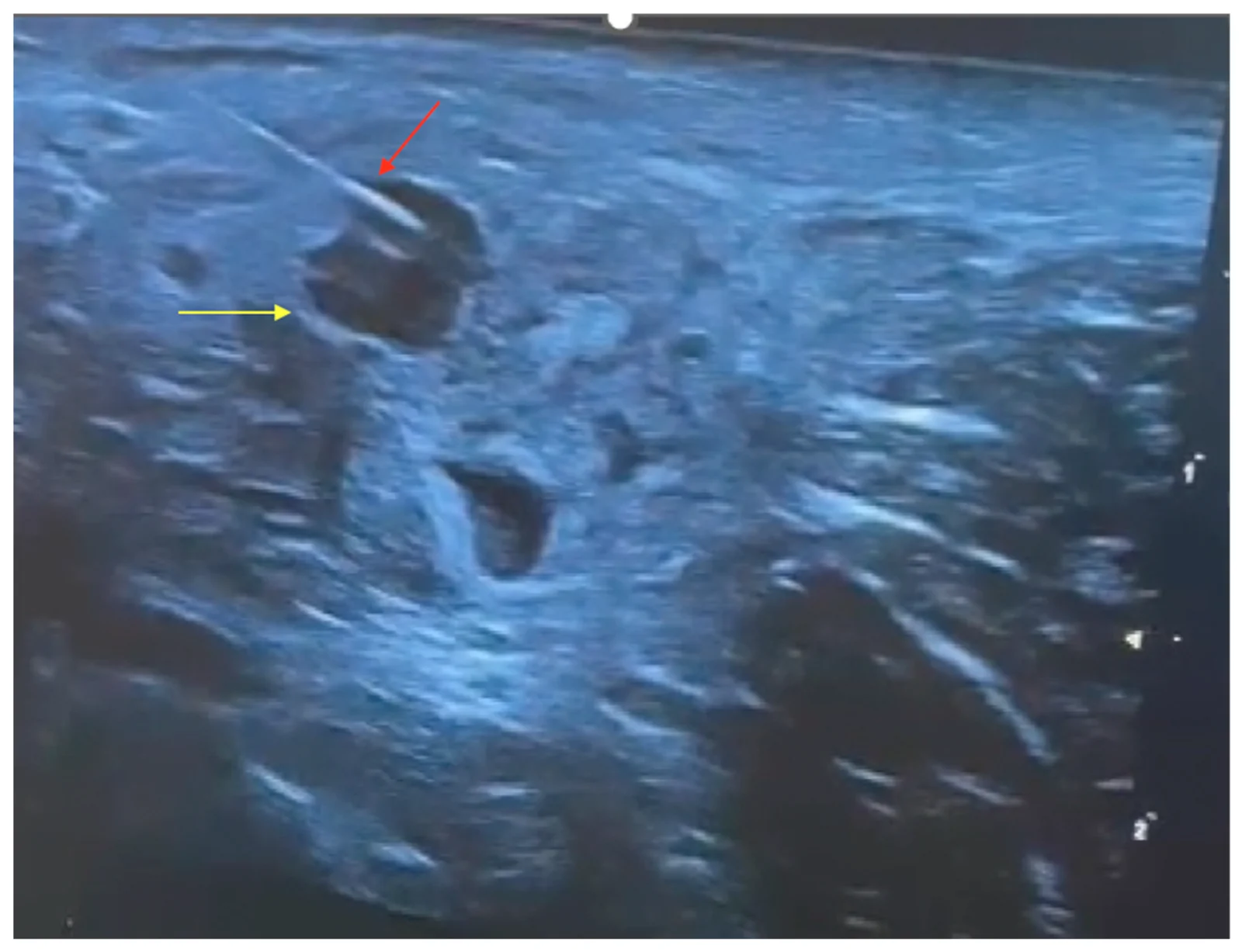
Ultrasound-guided needle aspiration biopsy of a laterocervical lymph node conglomerate composed of three adjacent lymph nodes. The largest node, measuring approximately 5 mm in short-axis diameter, was targeted for sampling. The needle tip (red arrow) is clearly visible within the superficial lymph node as well as the target lymph node (yellow arrow), confirming precise real-time ultrasound-guided tissue acquisition.

Notably, lymph node size was often small at the time of sampling. Twelve of twenty patients (60%) had laterocervical lymph nodes with a short-axis diameter of less than 15 mm. Among these, two patients had target lymph nodes with a short-axis diameter of only 5–6 mm (Figure 1 and Figure 2). Despite these small target dimensions, US-NAB provided adequate material for diagnostic evaluation in all cases, contributing to a definitive diagnosis in every patient included in the study.

Biopsy results are shown in Table 2. TB testing documented positivity for TB via PCR, culture, and bacterioscopic analyses in 45%, 15% and 5% of cases, respectively. Bacterioscopy and culture were not performed in 5/20 (25%) cases.

Histopathology demonstrated malignancy in 4/20 (20%) cases and non-malignant alternative diagnoses in 3/20 (15%) cases.

**Figure 3:**
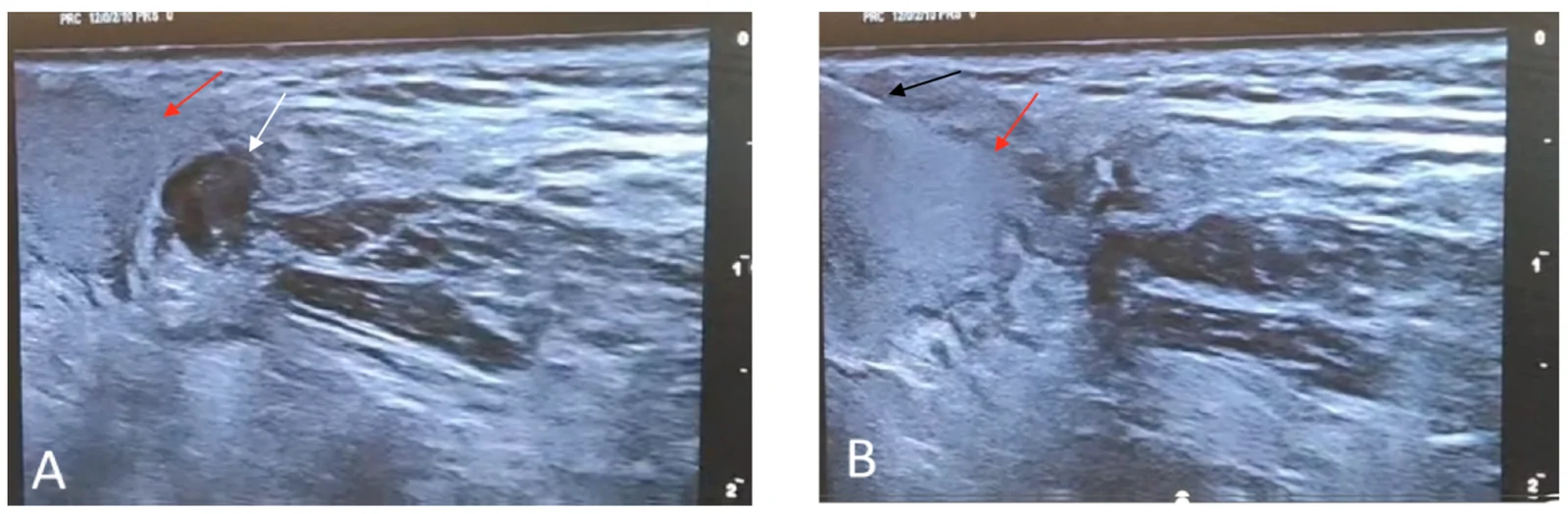
(**A**) Ultrasound image demonstrating scrofula, characterized by a heterogeneous subcutaneous collection overlying (red arrow) a small laterocervical lymph node (white arrow). (**B**) Ultrasound-guided sampling (black arrow) was performed both on the target lymph node and on the overlying heterogeneous collection. The aspirated material from the subcutaneous component tested positive for acid-fast bacilli (AFB), confirming tuberculous involvement.

Overall, a final diagnosis of tuberculous lymphadenitis was established in 13 of 20 patients (65%). Histopathological findings were consistent with TB in eight patients, supported by a positive QuantiFERON-TB test in four cases and a positive TB-PCR result in four cases. In the remaining five patients, histology demonstrated nonspecific necrosis; notably, all five specimens tested positive for TB-PCR, thereby establishing the diagnosis of tuberculosis. Adequate material contributing to a definitive diagnosis was obtained in all 20 procedures (20/20). Figure 1 and Figure 3 depict two representative cases of scrofula and their corresponding ultrasonographic features.

**Figure 4:**
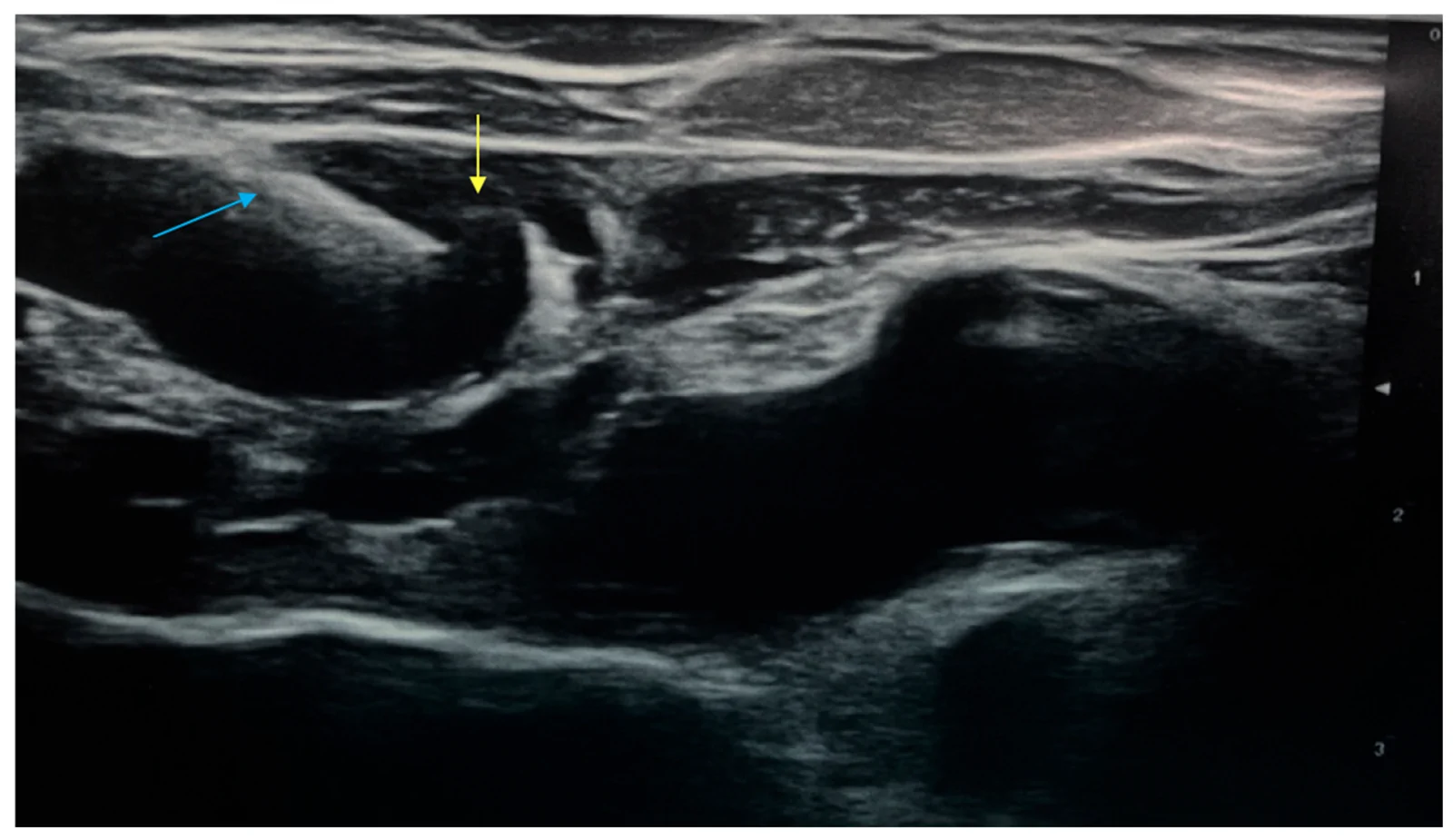
Ultrasound-guided needle aspiration biopsy (blue arrow) of a supraclavicular lymph node (yellow arrow) measuring approximately 12 mm in short-axis diameter. Histopathology showed non-necrotizing granulomatous inflammation, while QuantiFERON-TB, *Mycobacterium tuberculosis* PCR, and NTM-PCR were all negative. Final diagnosis: sarcoidosis.

**Figure 5:**
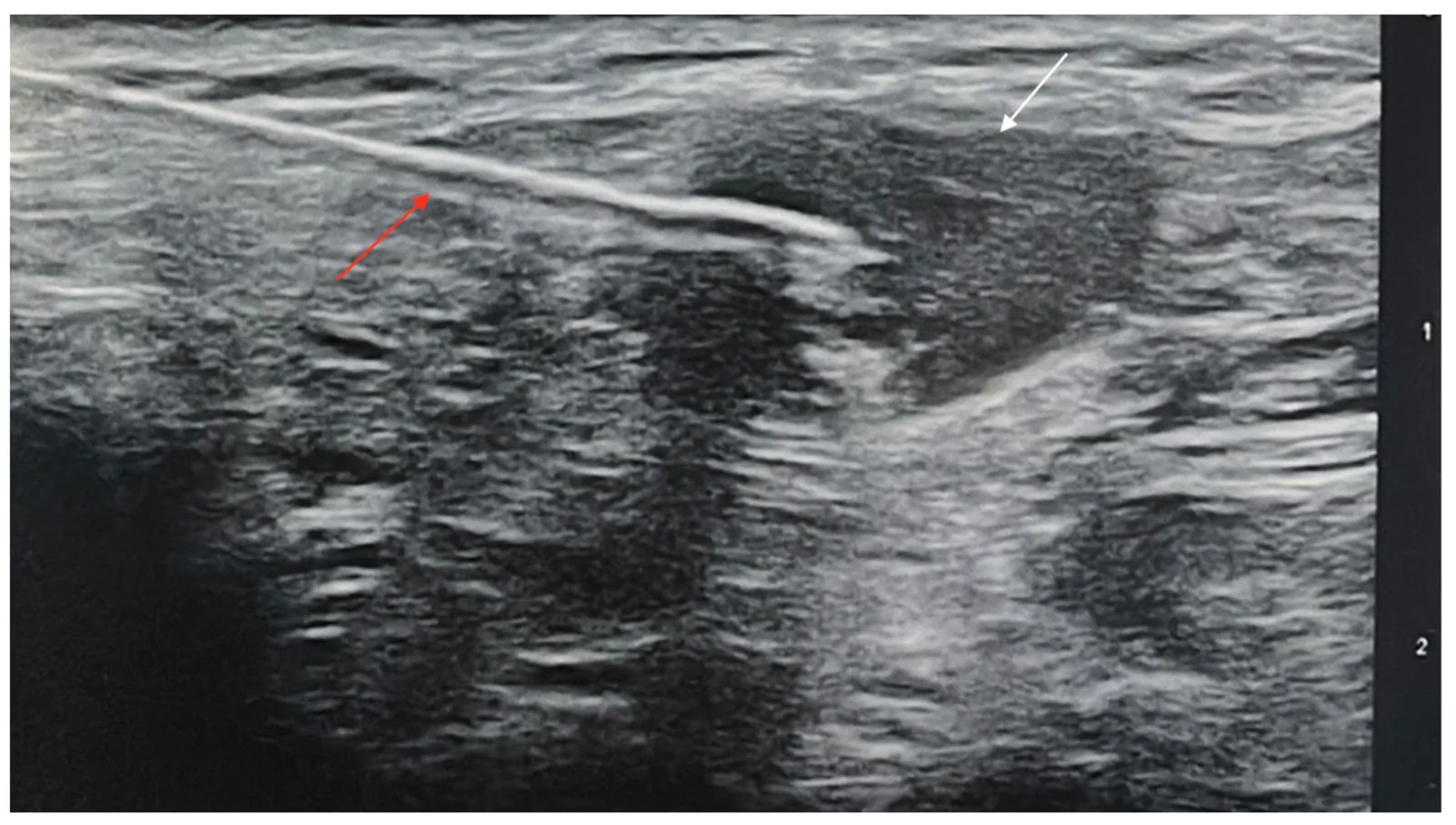
Ultrasound-guided needle aspiration biopsy of a heterogeneous laterocervical lymph node measuring approximately 12 mm in short-axis diameter in a patient with a positive QuantiFERON-TB assay. The lymph node lacked a clearly identifiable echogenic hilum (white arrow) and exhibited a markedly firm consistency, making needle advancement (red arrow) technically challenging during the procedure. Despite the initial suspicion of tuberculous lymphadenitis, histopathological analysis revealed metastatic lung adenocarcinoma, underscoring the importance of tissue sampling in the differential diagnosis of cervical lymphadenopathy.

A final diagnosis other than tuberculosis was established in 7 of 20 patients (35%), underscoring the diagnostic complexity of cervical lymphadenopathy and the importance of tissue sampling for differential diagnosis. Representative ultrasound images of two nontuberculous cases are presented in Figure 4 and Figure 5.

No major peri-procedural or post-procedural complications were recorded; specifically, there were no cases of bleeding, pneumothorax, or pneumomediastinum. No puncture-site hematoma or clinically relevant local pain was reported in the available medical records.

Patients diagnosed with tuberculous lymphadenitis were followed at our dedicated outpatient clinic for approximately 12 months. Patients diagnosed with sarcoidosis and bacterial lymphadenitis remained under clinical follow-up for more than 12 months. During the available follow-up, no diagnostic reclassification was observed, and none of the patients with an initial nontuberculous benign diagnosis were subsequently diagnosed with tuberculosis. Patients with malignant diagnoses were referred to a different specialist oncology center and therefore did not undergo systematic longitudinal follow-up at our institution.

## 4. Discussion

To date, TB is still a global health challenge, particularly in low- and middle-income countries, where limited resources and persistent socio-economic inequalities contribute to its ongoing burden [13]. *Mycobacterium tuberculosis*, the causative agent of TB, is an acid-alcohol-fast bacillus with a marked tropism for lymph nodes, which serve as key sites for its dissemination throughout the body [14]. In particular, these lymphatic niches also facilitate bacterial persistence due to the low tissue penetration of anti-TB drugs [14]. Hence, EPTB is one of the most insidious forms of TB and requires careful evaluation, given the wide array of differential diagnoses associated with this clinical presentation.

Ultrasound-guided core needle biopsy (US-CNB) has become increasingly adopted in clinical practice owing to advances in ultrasound technology and its ability to provide intact tissue cores for histopathological evaluation [15]. In contrast, our study describes the use of US-NAB with an 18-gauge modified Menghini aspiration needle, allowing tissue acquisition for histopathological, microbiological, and molecular analyses. In our cohort, adequate diagnostic material was obtained in all 20 procedures, with a final diagnosis of tuberculosis established in 13 patients and alternative diagnoses identified in the remaining seven.

Although Meng et al. investigated ultrasound-guided core needle biopsy rather than aspiration biopsy, their findings support the concept that ultrasound-guided tissue acquisition combined with molecular testing substantially improves the diagnosis of tuberculous lymphadenitis [8]. In particular, the combined use of histopathology and Xpert MTB/RIF on US-CNB samples achieved high diagnostic performance, yielding an area under the curve (AUC) of 0.956 on receiver operating characteristics (ROC) analysis, with a 70.1% sensitivity and a 100% specificity in diagnosing lymph node TB [8]. Although our small descriptive cohort does not allow a direct comparison of diagnostic accuracy with these data, adequate specimens contributing to a definitive diagnosis were obtained in all procedures using US-NAB.

Similarly, in a separate study, Yan et al. reported that combining US-CNB chest-wall biopsy with Xpert MTB/RIF achieved the highest diagnostic performance, with a sensitivity of 100% and a specificity of 94% [16]. This combined approach outperformed individual diagnostic methods, including acid-fast staining, mycobacterial culture, histopathology, and standalone Xpert, as well as paired combinations such as histopathology with acid-fast staining or culture [16]. Therefore, US-CNB may serve as a promising adjunct to existing diagnostic strategies, helping to overcome the well-known challenges associated with diagnosing lymph node TB.

In contrast, US-NAB also revealed other clinically relevant conditions in patients evaluated for suspected TB. Specifically, necrosis and malignancy were identified in 25% and 20% of cases, respectively. As with TB, these results are in line with the findings of Wang et al., who performed a retrospective study on 54 patients who received US-CNB combined with positron emission tomography-computed tomography on supraclavicular lymph nodes under the suspicion of cancer [17]. Their study provided not only an excellent overall diagnostic yield (98.1%) but also a high detection rate of malignancies (96.2%) [17].

Okasha et al. highlighted the important role of image-guided tissue acquisition combined with molecular diagnostics in the evaluation of tuberculous lymphadenopathy [9]. In their review, endoscopic ultrasound-guided tissue sampling demonstrated diagnostic yields ranging from 78% to 92% across different anatomical sites, while the integration of molecular techniques such as PCR and GeneXpert MTB/RIF further improved diagnostic accuracy and facilitated rapid diagnosis [11]. The authors concluded that ultrasound-guided sampling coupled with microbiological and molecular testing represents an effective strategy for overcoming the limitations of conventional diagnostic methods in tuberculous lymphadenopathy [11].

Our findings extend this concept to superficial cervical lymphadenopathy using a different sampling technique. Unlike the endoscopic ultrasound-guided tissue acquisition or ultrasound-guided core needle biopsy described in previous studies, we employed US-NAB using a modified Menghini aspiration needle. Despite relying on aspiration rather than tissue coring, this technique provided sufficient material for the diagnostic investigations performed, including histopathological, microbiological, and molecular analyses, and contributed to a definitive diagnosis in every patient included in our cohort.

These findings suggest that, when performed under real-time ultrasound guidance in easily accessible superficial cervical lymph nodes, US-NAB provides sufficient material for histopathological, microbiological, and molecular evaluation while offering a minimally invasive and readily available sampling approach. Rather than replacing core needle biopsy, US-NAB may represent a complementary sampling option, particularly in selected superficial lymph nodes, including very small targets, when adequate material for multimodal analysis can be obtained.

In this context, the recent dual-center retrospective study by Liu et al. [18], including 290 patients undergoing ultrasound-guided biopsy of cervical level IV lymph nodes, provides relevant comparative data on the use of modified Menghini needles for cervical lymph node sampling. The authors reported a sample adequacy rate of 94.9% (167/176) with the modified Menghini needle, comparable to that achieved with semiautomatic side-cut needles (93.0%, 106/114). Although their study primarily focused on histopathological sample adequacy and technical performance, these findings complement our observations. Our study addresses a different clinical setting, focusing on patients with suspected tuberculous lymphadenopathy and on the use of the obtained material for combined histopathological, microbiological, and molecular investigations, including sampling of very small superficial lymph nodes.

Regarding procedural safety, no major procedure-related complications were observed in our cohort. This finding is consistent with the low complication rates reported by Liu et al. and with previous studies evaluating ultrasound-guided needle aspiration biopsy of superficial metastatic lesions from lung cancer [18,19,20].

Beyond procedural safety, US-NAB proved valuable in the differential diagnosis of cervical lymphadenopathy. Tuberculosis has long been recognized as a “diagnostic chameleon”, as its clinical presentation and imaging findings frequently overlap with those of malignant and inflammatory diseases [21]. Constitutional symptoms, necrotic lymph nodes, and hypermetabolic lesions on imaging may closely resemble lymphoma or metastatic cancer, often leading to diagnostic uncertainty [21]. Consistent with this observation, more than one-third of patients in our cohort ultimately received a diagnosis other than tuberculosis, including malignant disease. These findings emphasize the importance of obtaining tissue confirmation whenever feasible and further highlight the pivotal role of ultrasound-guided sampling in establishing an accurate diagnosis, minimizing diagnostic delays, and enabling the timely initiation of the most appropriate treatment strategy.

Several limitations should be acknowledged. First, this was a retrospective, single-center study including a limited number of patients referred to a tertiary center for suspected tuberculous lymphadenopathy. Therefore, the observed sample adequacy cannot be interpreted as a formal estimate of sensitivity, specificity, or negative predictive value and may not be generalizable to other clinical settings. Second, procedures were performed by physicians experienced in ultrasound-guided interventions, and operator expertise may have contributed to the high sample adequacy observed, particularly when very small lymph nodes were targeted. Third, the study did not include a direct comparison with FNAC, US-CNB, or surgical biopsy, and no formal cost-effectiveness analysis was performed. Accordingly, our findings should primarily be interpreted as a description of technical feasibility and diagnostic contribution in a selected cohort. In addition, mycobacterial culture was not performed in five patients for organizational reasons. Therefore, although US-NAB provided material contributing to a definitive diagnosis in all cases, specimen adequacy specifically for culture-based mycobacterial diagnosis could not be assessed across the entire cohort.

Finally, clinical follow-up was retrospectively assessed as part of routine care and was not standardized according to a predefined study protocol. Patients with tuberculosis, sarcoidosis, and bacterial lymphadenitis were followed at our center for approximately 12 months or more without diagnostic reclassification. However, systematic longitudinal follow-up was not available at our institution for patients with malignant diagnoses, who were subsequently referred to another specialist center. Therefore, subsequent diagnostic reclassification cannot be completely excluded for the entire cohort. Larger prospective comparative studies are needed to establish the relative performance of US-NAB.

## 5. Conclusions

In this single-center cohort, ultrasound-guided needle aspiration biopsy (US-NAB) of laterocervical lymph nodes was feasible with no major procedure-related complications observed and provided clinically meaningful diagnostic information in patients with suspected tuberculous lymphadenitis. In our cohort, a definitive diagnosis was achieved in all patients, showing that aspiration-based sampling can provide clinically meaningful diagnostic information and adequate material for multimodal analysis.

An additional observation from our series was the technical feasibility of sampling lymph nodes with a short-axis diameter as small as 5–6 mm under continuous real-time ultrasound guidance. Although this finding requires confirmation in larger series, it illustrates the potential of the technique for tissue acquisition from very small superficial targets. Beyond confirming TB, US-NAB enabled the identification of alternative diagnoses, including malignant disease, highlighting its value in the differential diagnosis of cervical lymphadenopathy. This finding is particularly relevant given the well-recognized ability of TB to mimic neoplastic conditions both clinically and radiologically, making tissue confirmation essential for appropriate patient management.

Overall, our findings suggest that US-NAB may represent a practical sampling option for selected superficial and easily accessible cervical lymph nodes. Of particular interest, adequate tissue acquisition was achieved even from very small targets, supporting the technical feasibility of this aspiration-based approach under continuous real-time ultrasound guidance. Beyond tuberculosis, US-NAB also contributed to the identification of alternative benign and malignant diagnoses, highlighting its potential value within the diagnostic work-up of cervical lymphadenopathy. Larger prospective and comparative studies are warranted to better define its diagnostic performance relative to other tissue-acquisition techniques.

## Figures and Tables

**Table 1:** Demographic data collected at the time of the procedure.

Demographic Data	General Population *N* = 20	TB *N* = 13	Any Alternative Diagnosis *N* = 7
**Sex**			
- Female	11 (55%)	8 (62%)	3 (43%)
- Male	9 (45%)	5 (38%)	4 (57%)
**Age, years,** median [IQR]	46.5 [38.5–58]	42 [33–73]	52 [45–53]
**QuantiFERON-TB test:** Positive *n*/*N* (%)	14/18 (77.8%)	11 (85%)	3 (43%)
**Comorbidities:** Yes	11 (55%)	8 (62%)	3 (43%)
Hypertension	3 (15%)	2 (15%)	1 (14%)
Type 2 diabetes	2 (10%)	2 (15%)	/
Rheumatoid arthritis	2 (10%)	1 (8%)	1 (14%)
Alcoholism	1 (5%)	/	1 (14%)
Chronic urticaria	1 (5%)	1 (8%)	/
Pulmonary microembolism	1 (5%)	1 (8%)	/
Pituitary adenoma	1 (5%)	1 (8%)	/
**Immunocompromised:** Yes	1 (5%)	1 (8%)	0 (0%)
**Endemic countries**			
Yes	14 (70%)	10 (77%)	4 (57%)
No	6 (30%)	3 (23%)	3 (43%)
**Node dimension**			
- Short axis >15 mm	8 (40%)	7 (54%)	1 (14%)
- Short axis 8–15 mm	10 (50%)	4 (31%)	6 (86%)
- Short axis 5–7 mm	2 (10%)	2 (15%)	0 (0%)

**Table 2:** Biopsy results.

**TB Testing**		
**Needle TB** **PCR**	Positive	9 (45%)
	Negative	11 (55%)
**Needle TB** **Bacterioscopy**	Positive	1 (5%)
	Negative	14 (70%)
	Not performed	5 (25%)
**Needle TB** **Culture**	Positive	3 (15%)
	Negative	12 (60%)
	Not performed	5 (25%)
**Histopathology**		
**Consistent with TB** *n* = 8 (40%)	Necrotizing granulomatous inflammation with multinucleated giant cells	4 (20%)
	Non-necrotizing granulomatous inflammation with Langhans-type multinucleated giant cells	2 (10%)
	Caseous necrosis	2 (10%)
**Nonspecific histopathological findings** *n* = 5 (25%)	Necrosis (with TB DNA-PCR positive)	5 (25%)
**Malignancy** *n* = 4 (20%)	Lung adenocarcinoma	2 (10%)
	Pancreatic adenocarcinoma	1 (5%)
	Thymic carcinoma	1 (5%)
**Non-malignant alternative diagnoses** *n* = 3 (15%)	Sarcoidosis	2 (10%)
	Bacterial lymphadenitis	1 (5%)
**Final Diagnosis**		
**TB**		13 (65%)
**Any alternative diagnosis**		7 (35%)

*n* = 20, TB = tuberculosis; PCR = polymerase chain reaction.

## Data Availability

All relevant data are within the manuscript. Raw data are accessible, if requested, from the National Institute for Infectious Diseases “L. Spallanzani” Library at the following e-mail address: biblioteca@inmi.it.
